# Effects of Dry Needling of the Obliquus Capitis Inferior in Patients with Cervicogenic Headache and Upper Cervical Dysfunction: An Exploratory Randomized Sham-Controlled Trial

**DOI:** 10.3390/jcm14186619

**Published:** 2025-09-19

**Authors:** Marjolein Chys, Kayleigh De Meulemeester, Indra De Greef, Maxim De Sloovere, Barbara Cagnie

**Affiliations:** Department of Rehabilitation Sciences and Physiotherapy, Ghent University, Corneel Heymanslaan 10 (3B3), 9000 Ghent, Belgium; kayleigh.demeulemeester@ugent.be (K.D.M.); indra.degreef@ugent.be (I.D.G.); maxim.desloovere@ugent.be (M.D.S.); barbara.cagnie@ugent.be (B.C.)

**Keywords:** cervicogenic headache, dry needling, flexion–rotation test, obliquus capitis inferior

## Abstract

**Background/Objectives**: Cervicogenic headache (CeH) is linked to upper cervical dysfunctions. The obliquus capitis inferior (OCI) muscle may contribute to restricted cervical rotation at the C1–C2 level, altered proprioception and pain. Dry needling (DN) of the OCI is hypothesized to target these dysfunctions. The aim of this study was to investigate whether a single intervention combining DN and manual therapy (MT) compared to sham needling (SN) and MT, improves C1–C2 rotation, functional, headache-related and psychological outcomes in a subgroup of CeH patients with a positive cervical flexion–rotation test (CFRT). **Methods**: Thirty-four participants were randomly assigned to (1) DN or (2) SN. The primary outcome was C1–C2 rotational mobility. Secondary outcomes included headache-related parameters (frequency, intensity, duration and perceived effect), functional parameters (cervical mobility, pain pressure thresholds, motor control and proprioception) and psychological parameters (central sensitization, pain catastrophizing, coping strategies and kinesiophobia). Outcomes were re-evaluated at one-week follow-up. **Results**: Linear mixed-effects models showed a significant and clinically relevant increase of C1–C2 rotation in the DN group compared to the SN group post-intervention (mean difference [MD]: 4.51°; 95% confidence interval [CI]: 1.74; 7.28), which was maintained at the 1-week follow-up (MD: 5.44°; 95% CI: 2.55; 8.33). No clinically relevant changes were observed in other secondary outcome measures. **Conclusions**: Targeting the OCI may be of added value in restoring atlanto-axial dysfunction. While short-term mobility gains were observed, a single intervention appears insufficient as a stand-alone treatment to impact functional or psychological outcomes. Future research involving larger samples should examine DN effects as part of a multimodal approach with long-term follow-up.

## 1. Introduction

Headache disorders are common and cause substantial disability worldwide. Tension-type headache (TTH) and migraine are the most common recurrent headaches, with prevalence rates of 26% and 14%, respectively [1,2]. In contrast, the prevalence estimates of CeH are considerably lower, ranging from <1.0% to 4.1% in the general population, although it may also be prevalent in up to 18% of patients with frequent headaches [3,4]. The International Classification of Headache Disorders (ICHD) defines CeH as “Headache caused by a disorder of the cervical spine and its component bony, disc and/or soft tissue elements, usually but not invariably accompanied by neck pain (NP)” [5].

Cervicogenic headache is diagnosed based on clinical criteria such as unilateral headache without side shift, symptoms triggered by neck movement or sustained awkward positions and associated NP. These criteria can sometimes be doubtful due to symptom overlap with other headache types, like migraine or TTH [6,7,8]. In a Delphi study of Luedtke et al., the most clinically useful physical examination tests for the assessment of patients with headache were identified [9]. The majority of experts (>90%) indicated the CFRT as (extremely) useful for patients with CeH. Therefore, the CFRT is widely used in the assessment of upper cervical spine mobility impairments at the atlanto-axial level and in the diagnosis of CeH by physical therapists [9]. Studies have demonstrated that the CFRT is significantly reduced in patients with CeH compared to healthy individuals or patients with migraine and it is negatively correlated with headache intensity [6,10].

The CFRT was originally designed as an articular test to specifically evaluate the C1–C2 rotational mobility, since the C1–C2 rotation accounts for up to 60–70% of overall cervical axial rotation [11]. However, it is hypothesized that restricted atlanto-axial rotation may also result from tightness in the OCI muscle, which plays a key role in C1–C2 rotation due to its anatomical position [12,13]. Therefore, tightness in the OCI may also contribute to a positive CFRT. Additionally, the OCI has a high density of muscle spindles, which not only allow flexible movement, but also act as specific sensory receptors, making the OCI highly responsible for proprioception and the accurate positioning of the head and neck [14,15,16]. Additionally, studies showed that the OCI influences dural tension monitoring via a soft tissue myodural bridge, and it has been suggested that myodural bridges play a role in conditions resulting from excess dural tension, such as cervicogenic headaches [16,17]. Since the dura mater is innervated by the upper cervical nerves, pain arising from the cervical dura due to excess tension may be referred through the trigeminal nerve distribution and the convergence of upper cervical segment nociceptive afferents in the trigeminocervical complex [16,18]. This complex receives converging input from trigeminal afferents and afferents from the C1 to C3 spinal nerves and provides an anatomical basis for both headaches and neck pain to co-exist [19]. Since muscle tightness and the presence of local and referred pain may coexist with nociceptive input from myofascial trigger points, myofascial techniques have been studied more over the past decade.

Myofascial pain and dysfunction have been shown to be successfully treated by means of myofascial techniques such as manual techniques and DN. Although DN has been shown to effectively reduce pain and improve cervical mobility in patients with different types of headaches compared to no or placebo treatment [20,21], its specific added value alongside the well-established effects of MT in patients with CeH remains underexplored. To date, little is known about the combined mechanistic effect of MT and DN on the upper cervical region. Previous research already showed preliminary, positive treatment effects of needling the OCI by reducing cervical joint position error (JPE) and improving range of motion (ROM) in patients with cervicogenic vertigo, NP and headache [20,22,23]. However, there is no scientific evidence on the combined effects of DN and MT on functional and psychological outcome measures in patients with CeH.

The primary aim of this study is to evaluate the effect of DN of the OCI, combined with MT, for improving C1–C2 rotational mobility in a sample of CeH patients with a C1–C2 dysfunction, as identified by a positive CFRT. The secondary outcome measures comprise functional parameters (general mobility, pain pressure thresholds (PPT), motor control and proprioception), headache-related parameters (pain intensity, disability, impact on daily life and treatment effect) and psychological parameters (central sensitization, catastrophizing, coping and kinesiophobia).

## 2. Materials and Methods

### 2.1. Protocol and Registration

This study design was approved by the Ethics and Research Committee of Ghent University (project number BC-10474) and prospectively registered at Clinicaltrials.gov (registration number: NCT05074381). This trial was reported according to the STRICTA (extension of CONSORT for acupuncture studies) guidelines [24,25].

### 2.2. Study Design

This randomized, sham-controlled trial included patients presenting with headaches and NP. Participants were randomly allocated to a DN group or an SN group. Both groups received standardized MT and the outcome assessors were blinded to group allocation. Outcome measures, which were assessed at baseline, post-intervention and at the one-week follow-up, included functional, headache-related and psychological factors.

Between March 2022 and April 2025, patients presenting with headaches and neck pain were recruited. Recruitment took place via flyers in the waiting rooms of the Physical Medicine and Rehabilitation Department at Ghent University Hospital, as well as on social media. Before study inclusion, patients were asked to complete an online eligibility questionnaire about the current headache state, neck problems and general health. Participants were excluded if they had experienced trauma or surgery that could cause the headache, or if they had received treatment within the last month. More detailed inclusion and exclusion criteria are listed in Table 1. Possible eligible participants were invited to the baseline assessment for a clinical assessment to confirm full eligibility. Participants were included in this study if they had a positive CFRT (a 10-degree left–right difference in rotational ROM or a ROM of less than 32°) [26]. Eligible individuals were informed about the study procedures and provided written informed consent.

### 2.3. Randomization and Blinding

All procedures took place at the Department of Rehabilitation Sciences, Ghent University. An independent researcher randomly assigned participants to one of two study groups (DN or SN) using an internet-based randomization tool (www.randomizer.org) with a 1:1 allocation ratio. To ensure allocation concealment, sealed opaque envelopes were prepared and handled by an independent researcher. Participants were informed about the random group assignment, were blinded for treatment allocation and were therefore instructed not to reveal any treatment experience to their outcome assessment examiner. Due to the nature of the intervention, the therapist could not be blinded to the intervention. The outcome assessor was blinded to treatment allocation.

### 2.4. Sample Size

The sample size was determined using SPSS Statistics v.28 and calculated based on a significance level of 0.05 and a power of 80% to detect the minimal detectable change (MDC) of 7° in CFRT ROM based on previous data [27]. Following this calculation, 30 participants are required. We included 34 participants, accounting for a drop-out rate of 10%. The study was not designed or powered to detect statistically significant differences in secondary outcomes; analyses of these outcomes will therefore be considered exploratory and should be interpreted with caution.

### 2.5. Interventions

First, participants in both the DN and SN groups were manually treated with a solid filiform needle (0.30 × 0.40 mm C-Type acupuncture needle). Each participant was placed in a prone position with the arms comfortably supported in 90° shoulder abduction. The experimental group received DN of the OCI muscle, where the muscle is needled only in its medial half between the transverse process of C1 and the spinous process of C2 [28,29]. The needle was inserted perpendicular to the skin at a 45° angle between C2 and C1. This equates to directing the needle towards the patient’s opposite eye in a cranial-medial direction. To provide an additional reference point, the index finger of the opposite hand pointed to the contralateral eye, and the needle tip was directed towards the fingertip [28].

**Figure A1:**
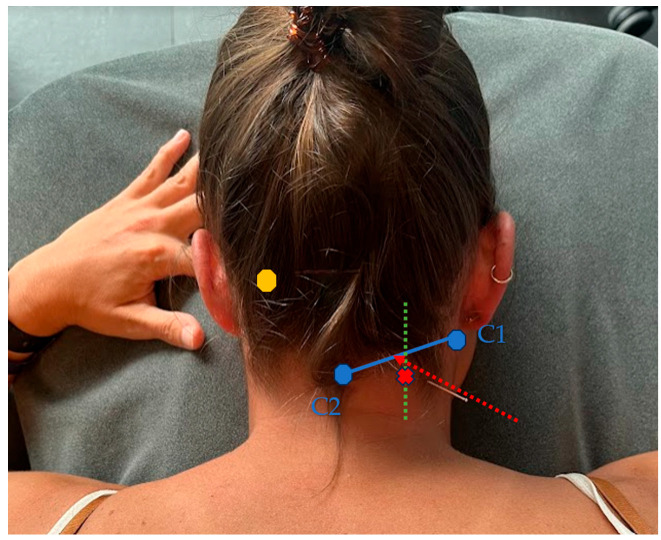
The participant was placed in a prone position with the arms comfortably supported in 90° shoulder abduction. The left index finger was positioned as an additional reference point (orange dot) at the corner of the left eye. The needle tip was angled towards the fingertip in a slight (10°) cranio-medial direction (red arrow, alongside the needle). The needle was inserted perpendicular to the skin (red cross) at a 45° angle between the processus spinosus of C2 and the processus transversus of C1 at the midzone between C2 and C1 (green line). The needle was advanced in a cranio-medial direction towards the anatomical location of the OCI until the tip of the needle reached the vertebral lamina of C2.

**Figure A2:**
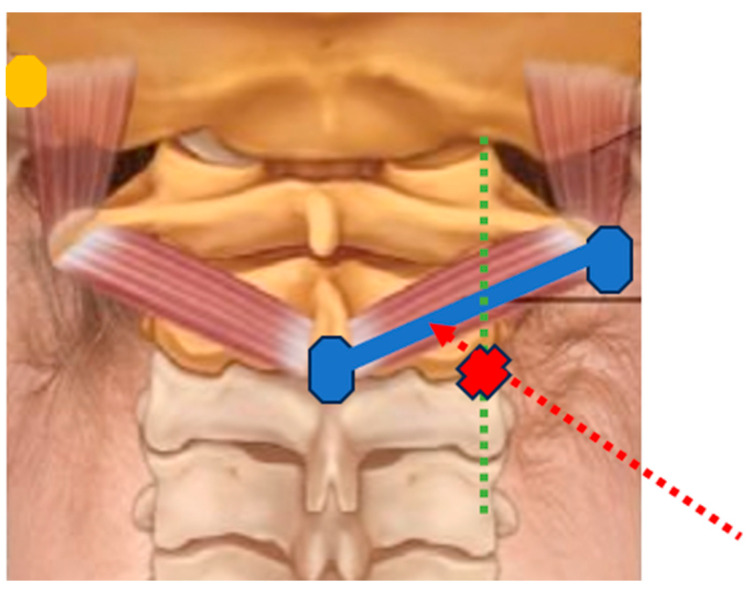
Schematic anatomical representation of the needling placement of the OCI.

The needle was advanced in a cranio-medial direction towards the anatomical location of the OCI until the tip of the needle reached the vertebral lamina of C2 (Appendix A, Figure A1 and Figure A2). The “fast-in and fast-out” or pistoning technique was used, whereby the needle was moved up and down (10 times) within the muscle, to provoke a local twitch response [30]. The control group received SN, whereby only the subcutaneous layer was punctured. The muscle fascia as well as the muscle itself remained unaffected with this technique. The same procedure was applied to simulate an authentic clinical experience and to preserve both credibility and participant blinding [31,32]. In the sham group, the needle was inserted into the subcutaneous layer at the trigger point location and moved up and down 10 times without penetrating the deep muscle fascia. As the fascia was not reached, no local twitch responses (LTRs) were elicited. Both the DN and SN groups received one insertion on each side. To ensure comparability, contextual elements typically associated with DN—such as skin disinfection, needle insertion and manipulation, and hemostatic compression—were performed identically in both interventions [31,32].

Secondly, both groups received the same MT intervention. A muscle energy technique (MET) with rotation on the atlantoaxial joint was performed as described by Fryer and Ruszkowski [33]. Additionally, a rotational sustained natural apophyseal glide (SNAG) was applied bilaterally to the C1–C2 level three times [34,35]. All interventions were performed by an experienced manual therapist with over 6 years of experience with DN and MT. Prior to the intervention, the therapist provided the same standardized information to all participants about the intervention and possible post-intervention effects.

### 2.6. Outcome Measures

All functional outcome measures were assessed at baseline, post-intervention and at 1-week follow-up. In addition, the CFRT was measured between the needling intervention and the MT intervention to assess the isolated and combined needling effect. Questionnaires were assessed twice, with a 1-week interval. Baseline and post-intervention measurements for each participant were performed by the same blinded assessor and verbal instructions were standardized for all participants. The sequence of the testing was randomly selected via the online tool Randomizer (www.randomizer.org). Adverse events were systematically questioned using two online questions at the 1-week follow-up.

#### 2.6.1. Primary Outcome Measure: C1–C2 Rotational Mobility

C1–C2 rotational mobility was assessed with the CFRT, using an EasyAngle digital goniometer (Meloq AB, Stockholm, Sweden). Participants were lying in a supine position, and the examiner initiated the procedure by gently positioning the participants’ neck in a fully flexed position and rotating their head to either side. The examiner would stop the rotation when they felt a moderate resistance or when the participants experienced discomfort or replication of headache symptoms. The test was conducted twice on each side, and the mean value of both repetitions was used for analysis. During the test, the EasyAngle was fixed on the participant’s head. This digital goniometer demonstrated a strong concurrent validity with the CROM device (ICC 0.97) and a good intra-rater reliability (ICC 0.94–0.96) [36].

#### 2.6.2. Secondary Outcome Measures

##### Functional Parameters

**Figure A3:**
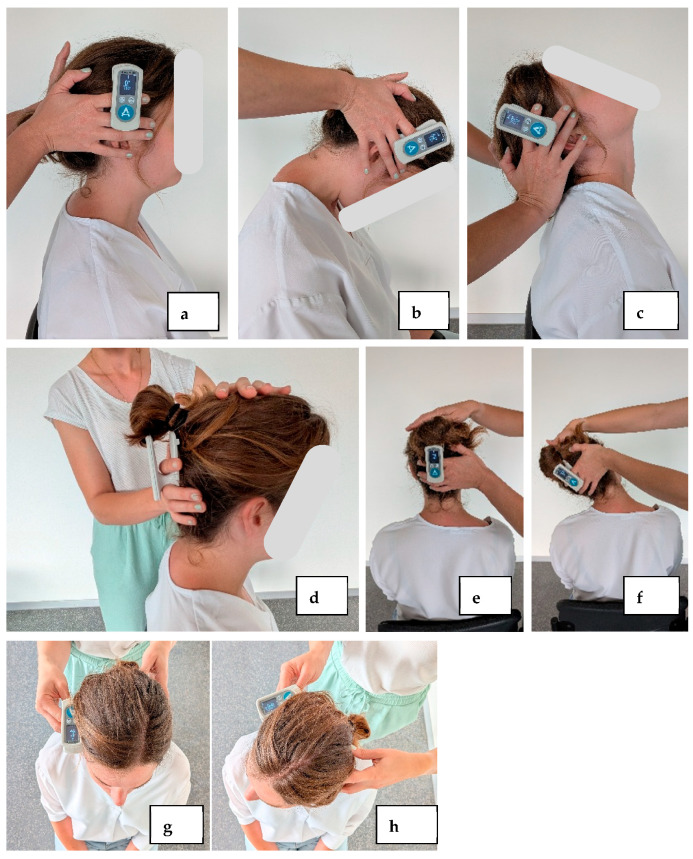
Starting position and measuring position of cervical flexion and extension (**a**–**c**), side bending (**d**–**f**) and rotation (**g**,**h**) in seated position.

Active ROM was also assessed using the EasyAngle. The patient’s active ROM for flexion, extension, rotation and side bending was assessed while the participant sat comfortably (back supported by the back of the chair, feet flat on the ground and knees, hips and ankles at 90°, and arms resting on the thighs). For flexion and extension, the EasyAngle was positioned vertically in front of the ear; for rotation, it was placed horizontally above the ipsilateral ear. For side bending, the base of the device was positioned posteriorly against the protuberantia occipitalis externa (Appendix B, Figure A3a–h). The patient was asked to perform these movements actively as far as tolerable. The average ROM of three trials was calculated and used for analysis.

The Cervical joint position error test (JPE) is used to evaluate cervical proprioception or kinesthesia [37]. Specifically, it measures the extent to which a person can accurately return the head to a central position following maximal rotation in the transversal plane [38]. Participants were sitting, eyes closed, 90 cm away from a wall with a lightweight laser-pointer frontally fixed through a headband [37,39]. Participants were instructed to slowly perform a full head rotation, minimizing vestibular input, and then return to the neutral position as precisely as possible, verbally indicating when they believed they had reached it. After each trial, the examiner manually repositioned the head to realign the laser pointer with the starting position. The discrepancy between the start and end positions was recorded in centimetres and subsequently converted to degrees [37,39]. Six trials to each side were performed and the mean was calculated to reduce the vulnerability to outliers [40,41]. A threshold of 4.5° was established as an abnormal repositioning value and significant for an impaired side (or both sides) [42]. A moderate to good (ICC 0.71) between-day reliability has been reported, and the MDC is −0.51° [43,44].

Pain pressure thresholds (PPTs) were measured with a hand-held pressure algometer (Wagner FPX 25 Force Gage). Measurements were taken bilaterally at the trapezius muscle at the midpoint between the C7 vertebra and the acromion. A third measurement was taken on the C2 processus spinosus. The probe (1 cm^2^) was placed perpendicular to the tested skin surface. Pressure was increased by 1 Newton(N)/s until the participant reported this feeling as unpleasant. This was repeated three times with a 30-s interval between each application. The average was calculated and used for analysis; the result was expressed in N/cm^2^. Digital algometry is shown to have almost perfect intrarater (ICC 0.82–0.94) and within-session test–retest (ICC 0.85–0.91) reliability [45]. The MDC ranged between 0.45 and 0.62 kg/cm^2^ in patients with chronic idiopathic NP [46].

Motor control was assessed using the cranio-cervical flexion test (CCFT). Both performance and endurance subscales were evaluated to assess the function of the deep cervical flexors [47]. A biofeedback pressure unit (Stabilizer; Chattanooga Group Inc.) was used and the subjects were placed in a supine position, with their heads on the inflatable unit and the gauge set at 20 mmHg. The participants were provided with instructions and were allowed a practice trial. The task involved a slight craniocervical flexion, like nodding. The subjects were then asked to limit each of the five progressive levels of the test within 10 s, without moving the gauge or using compensatory strategies. The CCFT showed moderate to good intra-rater (ICC 0.65) and inter-rater reliability (ICC 0.72) and a standard error of measurement less than 1.7 mmHg. The MDC was 4.60 to 4.81 [48,49,50,51].

##### Headache-Related Parameters: Pain Intensity, Disability, Impact on Daily Life and Treatment Effect

Pain intensity was assessed with the numeric pain rating scale (NPRS), using an 11-point scale, where 0 represents “no pain at all” and 10 “the worst possible pain”. The NPRS is considered a valid and reliable tool and has been shown to be reliable in people with mechanical neck complaints [52,53]. The MDC and minimal clinically important difference (MCID) are 2.1 and 1.3 points, respectively, in patients with mechanical neck pain [52].

Neck pain-related disability was assessed using the neck disability index (NDI), which measures self-reported pain intensity and limitations in performing daily work-related and non-work-related activities. The NDI indicates the extent to which neck pain interferes with daily life, and consists of 10 questions, scored (0–5) as a function of the impairment for each task. Total score ranges from 0 to 50. The NDI exhibits excellent test–retest reliability (ICC 0.88), the MDC was 6.9 points and the MCID varied from 5.5 to 10.5 points [52,53]. Additionally, the Headache Disability Inventory (HDI) investigates the impact of headaches on daily life [54]. Two additional questions examine the frequency and intensity of headaches. The questionnaire consists of a 25-item questionnaire with a maximum score of 100, which gives an impression of the self-perceived limitations due to headache. To determine whether the decrease in score is due to treatment effect, there must be at least a 29-point change [54]. Test–retest reliability ranges from 0.76 to 0.83 [54,55]. Impact on daily life was assessed with the Headache Impact Test (HIT-6). The questionnaire was developed to assess a broad spectrum of factors contributing to the burden of headaches and consists of 6 items. Scores range from 36 to 78, with higher scores representing greater disability [55,56]. The MCID of the HIT-6 is set at 8 points [57].

Treatment effect was assessed using the global perceived effect scale (GPES). This ordinal 7-point scale can be used as a self-report tool to report worsening or improvement of the patient’s pain condition. The GPES shows excellent test–retest reliability (ICC 0.90–0.99) [58].

##### Psychological Parameters

The central sensitization inventory (CSI) is a questionnaire used to assess the emotional and somatic symptoms commonly seen in central sensitization. A score ≥ 40/100 may indicate possible symptoms associated with central sensitization [59,60]. Scerbo et al. demonstrated that the use of CSI relates to high test–retest reliability (ICC 0.82–0.97) and moderate construct validity (0.63) [61].

The pain catastrophizing scale (PCS) is a self-assessment questionnaire to explore the level of catastrophizing. Three topics of catastrophizing are described: magnification, helplessness and rumination. A total score of 52 points can be calculated, with higher scores indicating more catastrophizing. The PCS has been shown to have adequate to excellent internal consistency reliability (ICC 0.83–0.93) [62].

The Tampa scale for kinesiophobia (TSK) is a patient-reported outcome measure developed to identify the fear of movement, fear of physical activity and fear avoidance. The total scores range from 17 to 68, with higher scores representing increased fear of movement. The TSK has shown good test–retest reliability (ICC 0.80) and excellent internal consistency (0.89) [63].

The pain coping inventory (PCI) consists of 33 items and is developed to evaluate the participants’ coping style, based on six cognitive and behavioural pain coping strategies [64]. The scale is dichotomized into two scores: reflecting an active or passive coping style. Test—retest stability ranged from 0.60 to 0.83. The criterion validity ranged from −0.30 to 0.65 [64].

### 2.7. Statistical Analysis

Data were analyzed according to the intention-to-treat principle using IBM SPSS Statistics version 29.0 (IBM, Armonk, NY, USA) for all outcome measures. Normality was assessed with the Shapiro–Wilk test and by visual inspection of histograms and Q–Q plots. Boxplots were used to identify outliers and extreme values. Means and standard deviations were calculated for demographic data. Linear mixed models (LMMs) were applied to examine within- and between-group differences over time for all primary and secondary outcomes. Fixed factors included “intervention” (DN vs. SN), “time” (baseline, post-intervention and 1-week follow-up) and the “intervention × time” interaction, with participants modelled as random intercepts. When significant main effects or interactions were observed, pairwise comparisons with Bonferroni adjustment were conducted. Change scores (relative to baseline) at post-intervention and 1-week follow-up were calculated to evaluate whether the minimal detectable change (MDC) was exceeded. Residuals were checked for normality, and all randomized participants were retained in the analyses, as LMMs estimate values for missing data [65] and their use is valid under the assumption that data are missing at random [66]. A generalized estimating equation (GEE) model with a cumulative logit link was used to assess changes in motor control performance and endurance over time between groups. Between-group effect sizes (ESs) were calculated using Cohen’s d: d = ((Mean_post_,DN − Mean_pre_,DN) − (Mean_post_,SN − Mean_pre_,SN))/(SD_pooled_). The effect was considered small (d = 0.2), medium (d = 0.5) or large (d = 0.8). Participants’ blinding was evaluated at 1-week follow-up and the Bang’s blinding index (BI) with a 2 (DN and SN) × 3 (DN, SN and do not know) format was calculated. Bootstrap resampling (n = 5000) was used to estimate 95% confidence intervals for the BI. Statistical significance was accepted at the 0.05 α-level.

## 3. Results

### 3.1. Participants

**Figure 1:**
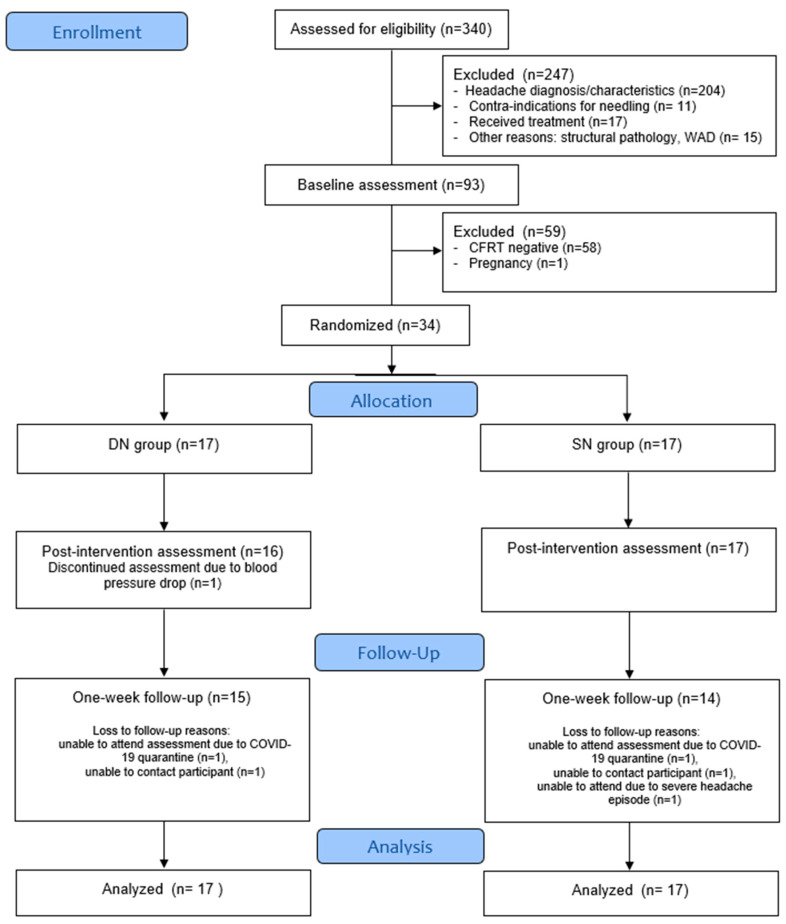
CONSORT flow diagram.

Of the 344 participants who completed the eligibility questionnaire, 93 were invited to participate in the baseline assessment. Based on eligibility screening during the clinical examination, 34 participants were included in the study and subsequently randomly allocated to the DN group (n = 17) or to the SN group (n = 17) (Figure 1). Demographic features of both groups are presented in Table 2. Patients’ characteristics between groups and outcome measures were comparable at baseline.

### 3.2. Primary Outcome Measure

**Figure 2:**
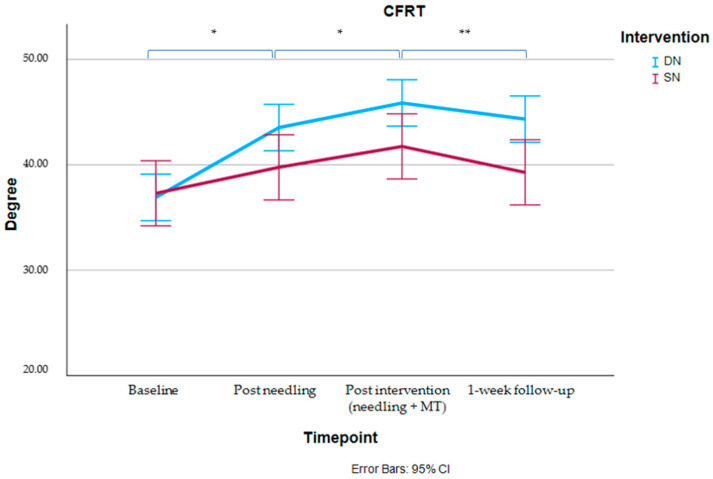
Between-group comparison for the CFRT at baseline and follow-up. Error bars: 95% CI. Post hoc: * p < 0.05; ** p < 0.001.

The LMM analysis showed a significant group-by-time interaction effect for the C1–C2 rotational mobility, measured by the CFRT (*p* = 0.001), indicating a greater increase in the CFRT in the DN group compared to the SN group post needling (MD: 4.15°; 95% CI: 1.14; 6.89), post-intervention (MD: 4.51°; 95% CI: 1.74; 7.28) and at 1-week follow-up (MD: 5.44°; 95% CI: 2.55; 8.33), compared to baseline (Table 3 and Figure 2).

### 3.3. Secondary Outcome Measures

#### 3.3.1. Functional Outcome Measures

For global active ROM, a group-by-time interaction was found for cervical flexion (*p* = 0.024), showing a significant increase in active flexion for the DN group, compared to the SN group at one-week follow-up, compared to baseline (MD: 6.08°; 95% CI: −0.044; 2.20). No group-by-time interaction effect was found for extension (*p* = 0.074), side bending (*p* = 0.905) or rotation (*p* = 0.764) and main effects for time and group remained above the significance level (Table 4).

Similarly, no group-by-time interaction was seen for the PPTs at the upper trapezius and C2 level (*p* = 0.620 and *p* = 0.540, respectively). No main effect for time or group was found.

Considering the JPE, 41.2% of the participants in the DN and 41.2% in the SN group had a positive test (>4.5° deviation) at baseline, while at one-week follow-up, this was 17.6% and 29.4% in the DN and SN group, respectively. There was no group-by-time interaction when comparing the proportion of participants with a positive test (GEE: *p* = 0.145). There was no group-by-time interaction for JPE change in deviation (*p* = 0.413). However, there was a significant main effect for time (*p* = 0.049); with a clinically relevant reduction in the SN group from baseline to 1-week follow-up (DN: MD: −0.42°; 95% CI: −1.21; 0.37 and SN: MD: −0.66°; 95% CI: −1.47; 0.15).

A significant group-by-time interaction for motor control performance was observed (Wald χ^2^(2) = 11.64, *p* = 0.003). There was also a significant main effect for time (Wald χ^2^(2) = 13.10, *p* = 0.001), but not for group (Wald χ^2^(1) = 1.14, *p* = 0.285). At baseline, the DN group demonstrated significantly higher CCFT performance compared to the SN group (B = 1.51; SE = 0.70, Wald χ^2^(1) = 4.70, *p* = 0.030). At 1-week follow-up, the SN group showed a pronounced improvement from baseline to follow-up (B = −2.40, SE = 0.72, Wald χ^2^(1) = 11.21, *p* < 0.001), whereas this improvement was substantially smaller for the DN group (B = −2.40, *p* < 0.001). No significant changes from baseline to post-intervention were observed in either group (B = −0.24, SE = 0.46, Wald χ^2^(1) = 0.27, *p* = 0.604). For the endurance subscale, the analysis revealed no significant interaction effect (Wald χ^2^(2) = 4.57, *p* = 0.102) or main effect for time (Wald χ^2^(2) = 1.94, *p* = 0.379), although there was a main effect for group (Wald χ^2^(1) = 4.58, *p* = 0.032).

#### 3.3.2. Headache-Related Outcome Measures

There was no time-by-group interaction for the average pain score (*p* = 0.540) or maximal pain score (*p* = 0.675), measured during the past 7 days. LMM showed similar reductions (MD: 0.49; 95% CI: −1.12; 2.09) for both groups, although not significant. The between-group difference for the GPES was also not statistically significant (*p* = 0.978) at 1-week follow-up, measured with the Mann–Whitney U test. There was no significant interaction effect for the NDI (*p* = 0.942), HIT-6 (*p* = 0.990) or HDI (*p* = 0.053). Post hoc pairwise comparisons showed a main effect for time for headache-related impact and disability, reflected in similar reductions in the scores of the HIT-6 (*p* < 0.001) for both groups and a reduction in the HDI (*p* = 0.031) in the SN group (MD: −6.76; 95% CI: −13.61; 0.086). (More detailed information is available in Supplementary Table S1).

#### 3.3.3. Psychological Outcome Measures

For the questionnaires, there was a significant interaction effect for the PCS (*p* = 0.042) with larger reductions in the SN group (MD: 7.22; 95% CI: 0.28; 14.16). No significant interaction effects were found for the CSI (*p* = 0.488) or TSK (*p* = 0.183). For the PCI, GEE showed a non-significant time-by-group interaction (χ^2^(1) = 0.32, *p* = 0.574). (More detailed information is available in Supplementary Table S1).

### 3.4. Adverse Events, Missing Data and Blinding

Four participants (two in the DN group and two in the SN group) reported muscular stiffness during the study course. Nine participants (five in the DN group, four in the SN group) reported headache and/or NP during the follow-up period of one week. Participants were assumed to be missing at random, and demographics and headache characteristics did not differ between groups. Bang’s blinding index (BI) was calculated for each group [67]. In the DN group, the BI was 0.32 (95% CI: −0.40 to 0.77), indicating a tendency toward correct guessing but not statistically significant. In the SN group, the BI was −0.29 (95% CI: −1.43 to 0.36), suggesting participants tended to guess incorrectly, although this was also not statistically significant. As both confidence intervals included zero, blinding was considered to be preserved (Appendix C, Table A1).

## 4. Discussion

This study provides additional insights into the mechanistic effects of DN on atlanto-axial dysfunction in CeH. The results offer partial support for the efficacy of DN, particularly in improving C1–C2 rotational mobility, while other outcomes showed limited or no differential effects between groups, although this might have been influenced by sample size.

A significant group-by-time interaction was observed for the CFRT, with the DN group demonstrating consistently greater improvements than the SN group across all post-intervention time points. The effect sizes were found to be large (>0.80), with the mean differences ranging from 4.15° immediately post-needling to 5.44° at one-week follow-up, all of which were statistically significant. The results suggest that DN may be effective for improving C1–C2 mobility, a key dysfunction in CeH. Both isolated and combined DN interventions led to gains in rotation, with only the DN group achieving statistically and clinically meaningful improvements exceeding the MDC of 7° immediately post-intervention. These gains were maintained at one week, although between-group differences did not surpass the MDC. The results also indicate that both DN and MT contributed to the observed improvements.

Additionally, the JPE test was utilized to evaluate proprioception. At one-week follow-up, the SN group exhibited a clinically relevant improvement. The DN group demonstrated a comparable reduction in JPE, although this remained below the threshold for clinical relevance. Notably, the baseline JPE was higher in the SN group, which may have influenced the observed differences, potentially due to a regression to the mean effect. A subsequent analysis of the number of participants with a positive JPE test at baseline revealed a downward trend in the frequency of positive JPE tests at one-week follow-up, although this was not statistically significant. These preliminary findings suggest a potential benefit that warrants further investigation with larger sample sizes.

Changes in global cervical ROM were limited, with significant improvements observed only in cervical flexion, but results did not exceed the MDC.

No statistically significant or clinically relevant alterations were observed in relation to other functional or headache-related outcomes, which may be related to the short follow-up duration, a low intervention dose and the multifactorial nature of CeH pathophysiology.

When comparing our results to other research, a study by Sillevis et al. demonstrated a clear link between the muscle’s functional role and positional fault by applying electrical muscle stimulation of the OCI. The study examined the position of the atlas and observed significant changes in muscle length that correlated with the palpated default position of the atlas [12]. These findings reinforce the idea that increased suboccipital muscle tone can contribute to a C1–C2 mobility dysfunction. This was confirmed by a study of Murillo et al. who demonstrated that a single DN session targeting the OCI led to short-term improvements in C1–C2 rotational mobility in patients with traumatic or idiopathic neck pain [13]. It was also shown that DN is useful to reduce disability related to cervical mobility deficits and that increasing cervical mobility mediates the needling effect on NP-related disability [68].

Murillo et al. also investigated the JPE and included patients only if they exhibited an impaired JPE in at least one direction, which was not a criterion in the current study. They showed that a single DN session targeting the OCI led to short-term improvements in proprioception as well [13].

Furthermore, the OCI contains the highest density of muscle spindles of all suboccipital muscles and should be regarded as a critical proprioceptive sensor, specialized in detecting and transmitting detailed information about joint position and movement [15]. Muscular dysfunction may result in altered proprioceptive feedback and other symptoms such as cervicogenic dizziness and somatosensory tinnitus [12,18,22,69,70,71]. In a case study evaluating the diagnostic value of DN for cervicogenic dizziness, three participants with clinical signs of the condition were included [22]. These signs included a positive CFRT, restricted cervical ROM and tenderness in the upper cervical region. Participants received DN targeting the OCI. Two participants reported complete symptom resolution, while the third experienced significant improvement [22]. These effects were sustained for at least six months, highlighting the potential of DN as both a diagnostic and therapeutic tool. Another case report reported that targeting the muscles of the upper cervical spine with DN resulted in a meaningful reduction in cervicogenic somatosensory tinnitus, and the improvements persisted at 1-year follow-up [70].

When looking at headache-related parameters, a meta-analysis of Kandeel et al. showed DN to be a potent intervention, reducing headache intensity and frequency, although with a lower impact on disability scores [21]. The number of studies on CeH was limited, as was the number of studies incorporating needling of the suboccipital region, so generalizability is limited. The observed benefits were especially pronounced after one and three months. In consideration of the specified timeframe, it is possible that the duration of the follow-up period in this study, in conjunction with the sample size, may be inadequate for the detection of alterations in secondary outcome measures.

Another review of Pourahmadi et al. showed that in CeH, for every three or four patients treated with DN, one patient will likely exhibit decreased headache intensity (NNT = 4; small effect) and improved related disability (NNT = 3; medium effect) [20]. Considering the heterogeneity within patients with headache, selecting patients with a dominant myofascial dysfunction might aid in optimal patient selection. Accordingly, the CFRT could be a marker that could define the usefulness of a given therapeutic technique; it might be useful for diagnosis as well as re-evaluation, and therefore, help the clinician to choose the most appropriate and tailored treatment options [72].

To date, only a few studies have assessed DN within multimodal care; Mousavi-Khatir et al. demonstrated that adding DN to a physical therapy program (comprising 15 sessions, of which 4 included DN) exerted a positive effect on headache intensity, headache frequency, associated disability, performance of the CCFT and cervical active ROM in patients with CeH presenting with active triggerpoints in the suboccipital, upper trapezius and sternocleidomastoid muscles. Nonetheless, the observed changes did not reach clinical relevance when compared to SN or an absence of needling intervention [73]. Additionally, a study by Patra et al. evaluated the effectiveness of DN and Mulligan C1–C2 SNAGs in increasing PPTs and reducing headache disability in patients with CeH [74]. There was a consistent reduction in tenderness and improvement in disability of the patients belonging to all groups, with larger improvements in the combined treatment group (DN + MT). The findings of this latter study suggest that the combination of DN and C1–C2 SNAGs is more advantageous for patients with CeH. The SNAG technique in the present study differed from the one utilized in the aforementioned study [74]. Consequently, reproducibility is challenging due to the absence of information regarding the applied intervention.

### 4.1. Strengths and Limitations

To our knowledge, this is the first controlled clinical study to investigate changes in C1–C2 rotation and headache-related parameters in individuals with CeH. The DN protocol follows a recently published guide, written by a group of international experts, to enhance reproducibility [28,75]. Secondly, maximal effort was conducted to obtain proper participant blinding: the therapist was mindful of potential confounding influences for sham procedures and emphasized the overall treatment experience, in line with current recommendations [31,32,76,77]. The BI showed acceptable results, indicating adequate blinding. Furthermore, participants were selected based on a positive CFRT, increasing subgroup homogeneity. Lastly, the EasyAngle device is a reliable and user-friendly digital inclinometer, offering practical and objective applicability in clinical settings [36]. A considerable limitation is that some study participants had prior experience with needling interventions, which could have influenced their cognitive processes, participant blinding and study outcomes despite the blinded group allocation. However, these participants were evenly distributed among the treatment groups. A second limitation is the limited statistical power for secondary outcomes. Furthermore, the short follow-up duration, low intervention dose, and the multifactorial nature of CeH pathophysiology may also have contributed to the absence of significant effects. Accordingly, findings related to secondary outcomes should be interpreted with caution, given the risk of both type I and type II errors. Additionally, the possibility of a type I error due to multiple testing cannot be excluded. As this was an exploratory study, no correction for multiple comparisons was applied, and further research is needed to confirm these findings. Moreover, the confidence intervals for mean differences were wide, indicating low precision of the effect estimates. Therefore, the results should be interpreted with caution. Additionally, generalizability to other headache forms is still unexplored, which limits the external validity of this trial. Lastly, the study focused only on immediate and short-term effects; no long-term follow-up was conducted to assess changes in functional, headache-related or psychological outcomes.

### 4.2. Implications for Clinical Practice and Future Research

Dry needling may serve as a useful technique to help restore rotational mobility at the C1–C2 segment. While the combination of DN and MT achieved the MCID, other outcome measures did not show significant changes and failed to reach clinically relevant thresholds. The clinical impact of this trial is therefore limited, as it did not lead to immediate reductions in pain or disability. Moreover, the intervention focused solely on a hands-on manual technique, which did not fully address the complex nature of CeH. In fact, a recent study of Mingels et al. explored the multidimensional characteristics of CeH, showing higher degrees of central sensitization, lower PPTs and a decreased sleep quality and headache-related quality of life compared to healthy controls. Also, significant relations between pain processing and (1) lifestyle characteristics and (2) psychosocial characteristics were observed in the CeH group, emphasizing the need for more tailored and multidimensional rehabilitation strategies [78].

Although DN and MT together reached the MCID, the lack of broader clinical improvements may be due to the limited sample size and follow-up in this exploratory trial. A follow-up duration of at least 3 months might be necessary to assess relevant changes [21]. In the event of contra-indications for DN, MT remains the gold standard treatment option. Additionally, the identification of potential biomarkers for patient selection may be a valuable strategy to optimize treatment responsiveness and tailor interventions more effectively. Overall, the findings suggest that a single intervention is insufficient, and DN targeting the OCI should not be used in isolation. Instead, it should be integrated into a comprehensive, multimodal, individualized treatment plan aimed at addressing headaches in a holistic manner.

## 5. Conclusions

A single session of DN of the OCI improves C1–C2 axial rotation, with a larger effect when DN is combined with MT. The observed effects remain at one-week follow-up; however, no clinically relevant changes in secondary outcome measures are found and long-term follow-up is necessary. Future studies should explore whether adding the technique to multimodal treatment improves headache management.

## Figures and Tables

**Table 1:** Eligibility criteria.

Inclusion Criteria	Exclusion Criteria
18–65 years Positive CFRT: right/left difference ≥ 10° in rotational ROM or unilateral rotational ROM ≤ 32° [26] Diagnosis of CeH, fulfilling the ICHD-3 criteria [5] Headache and neck pain for at least one day/week for a minimum of three months	Diagnosis of a primary headache (i.e., migraine, tension-type headache, cluster headache, …) Whiplash/post-traumatic headache History of head/neck/shoulder surgery, cervical/cranial nerve radiculopathy Treatment: DN, chiropractic, osteopathic, MT or a physiotherapeutic intervention in the past month or taking medication, influencing muscle tone. Contra-indications for DN: fear of needles, taking anticoagulants, coagulation disorders, pregnancy, epilepsy…

Abbreviations: CeH, cervicogenic headache; ICHD-3, The International Classification of Headache Disorders—version 3; CFRT, cervical flexion–rotation test; ROM, range of motion; NPRS, numeric pain rating scale.

**Table 2:** Patient characteristics at baseline.

	DN Group N = 17	SN Group N = 17
Age (y)	34.4 ± 11.4	38.6 ± 13.0
Sex; female/male (% female)	13/4 (76.5%)	13/4 (76.5%)
Length (cm)	171.5 ± 11.9	170.5 ± 9.4
Weight (kg)	71.6 ± 20.5	71.1 ± 14.0
BMI (kg/m^2^)	24.0 ± 4.0	24.6 ± 5.6
Educational level; n (%)		
Secondary school degree	4 (23.5%)	5 (29.4%)
College degree	7 (41.2%)	10 (58.8%)
University degree	6 (35.3%)	2 (11.8%)
Job; n (%)		
Student	3 (17.6%)	3 (17.6%)
Employed	11 (64.7%)	12 (70.6%)
Self-employed	2 (11.8%)	1 (5.9%)
Invalidity	1 (5.9%)	0 (0.0%)
Retired	0 (0.0%)	1 (5.9%)
Headache		
Intensity (past 7 days), NPRS (/10):		
- Average headache intensity	4.1 ± 1.9	5.6 ± 2.1
- Worst headache intensity	6.1 ± 1.8	7.7 ± 1.5
Frequency (days/month)	11.2 ± 7.6	12.2 ± 5.9
Self-reported severity; n (%)		
- Mild	0 (0.00%)	2 (11.8%)
- Moderate	11 (64.7%)	4 (23.5%)
- Severe	5 (29.4%)	9 (52.9%)
- Very severe	1 (5.90%)	2 (11.8%)
Lifestyle		
Pc-work (hrs/w)	24.6 ± 13.7	25.9 ± 21.3
Sport (hrs/w)	2.3 ± 1.5	2.7 ± 2.8
Sleep (hrs/night)	7.3 ± 0.9	7.0 ± 0.9
Sleep problems related to headache		
- Yes	11 (64.7%)	8 (47.1%)
- No	6 (35.3%)	9 (52.9%)

Data are mean ± standard deviation or frequency (proportion). Abbreviations: BMI, body mass index; NPRS, numeric pain rating scale; Pc, personal computer; Hrs, hours.

**Table 3:** Primary outcome measure.

C1–C2 Rotational Mobility; CFRT (°)	DN Group n = 17	SN Group n = 17	Between-Group Change Effect Size (ES)
Baseline	36.91 (1.48)	37.30 (1.48)	
Post needling	43.53 (1.48)	39.76 (1.48)	
Within-group change post needling to baseline	6.62 (3.99; 9.25) *	2.47 (−0.16; 5.10)	4.15 (1.14; 6.89) * ES: *d* = 1.03
Post manual therapy	45.86 (1.49)	41.74 (1.48)	
Within-group change post intervention to baseline	8.95 (6.26; 11.64) **	4.44 (1.81; 7.07) **	4.51 (1.74; 7.28) * ES: *d* = 1.11
1W follow-up	44.34 (1.51)	39.28 (1.53)	
Within-group change 1W follow-up to baseline	7.42 (4.68; 10.17) **	1.98 (−0.83; 4.79)	5.44 (2.55; 8.34) ** ES: *d* = 0.84

Abbreviations: W, week; DN, dry needling; MT, manual therapy; SN, sham needling; ES, effect size; *d*, Cohen’s *d*. * < 0.05; ** < 0.001.

**Table 4:** Secondary outcome measures.

	DN Group n = 17	SN Group n = 17	Between-Group Change Effect Size (ES)
Functional Parameters			
Global active ROM (°)			
FLEXION			
Baseline	54.69(2.88)	56.35 (2.88)	
Post intervention	51.39 (2.92)	55.47 (2.88)	
Within-group change post intervention to baseline	−3.29 (−8.45; 1.86)	−0.88 (−5.90; 4.14)	−2.41 (−8.26; 3.43)
1W follow-up	60.50 (2.96)	56.08 (2.99)	
Within-group change 1W follow-up to baseline	5.81 (0.54; 11.09) *	−0.27 (−5.66; 5.12)	6.08 (−0.044; 2.20)
EXTENSION			
Baseline	60.26 (3.02)	51.49 (3.02)	
Post intervention	60.64 (3.08)	50.31 (3.02)	
Within-group change post intervention to baseline	0.38 (−6.36; 7.13)	−1.18 (−7.76; 5.41)	1.56 (−6.09; 9.21)
1W follow-up	54.57 (3.14)	53.12 (3.19)	
Within-group change 1W follow-up to baseline	−5.68 (−12.58; 1.22)	1.63 (−5.42; 8.68)	−7.31 (−15.32; 0.70)
SIDE BENDING			
Baseline	41.54 (2.60)	37.03 (2.60)	
Post intervention	42.68 (2.66)	36.64 (2.60)	
Within-group change post intervention to baseline	1.14 (−5.04; 7.32)	−0.39 (−6.43; 5.65)	1.53 (−5.48; 8.55)
1W follow-up	42.19 (2.77)	37.22 (2.76)	
Within-group change 1W follow-up to baseline	0.65 (−5.82; 7.13)	0.19 (−6.27; 6.65)	0.46 (−6.96; 7.89)
ROTATION			
Baseline	67.51 (2.86)	60.26 (2.91)	
Post intervention	69.43 (2.86)	64.31 (2.86)	
Within-group change post intervention to baseline	1.92 (−4.11; 7.94)	4.06 (−1.82; 9.94)	−2.14 (−8.98; 4.69)
1W follow-up	67.99 (2.96)	60.60 (3.01)	
Within-group change 1W follow-up to baseline	0.48 (−5.69; 6.64)	0.34 (−5.96; 6.64)	0.14 (−7.02; 7.29)
Joint position error (°)			
Baseline	3.55 (0.28)	3.96 (0.28)	
Post intervention	3.30 (0.28)	4.07 (0.28)	
Within-group change post intervention to baseline	−0.25 (−1.00; 0.51)	0.12 (−0.64; 0.87)	−0.36 (−1.23; 0.51)
1W follow-up	3.13 (0.29)	3.30 (0.30)	
Within-group change 1W follow-up to baseline	−0.42 (−1.21; 0.37)	−0.66 (−1.47; 0.15)	0.24 (−0.67; 1.16)
Joint position error (% of participants with a restriction)			
Baseline; n (%)	7/17 (41.2%)	7/17 (41.2%)	
Post intervention; n (%)	4/17 (23.5%)	10/17 (58.8%)	
1W follow-up; n (%)	3/15 (20%)	5/14 (35.7%)	
Pain pressure threshold			
UPPER TRAPEZIUS (N/cm^2^)			
Baseline	21.34 (3.86)	24.47 (3.86)	
Post intervention	22.18 (3.89)	23.00 (3.86)	
Within-group change post intervention to baseline	0.84 (−3.83; 5.52)	−1.47 (−6.01; 3.07)	2.31 (−2.98; 7.60)
1W follow-up	22.06 (3.91)	25.12 (3.93)	
Within-group change 1W follow-up to baseline	0.72 (−4.06; 5.51)	0.72 (4.06; 5.51)	0.068 (−5.48; 5.62)
C2 (N/cm^2^)			
Baseline	18.39 (3.22)	19.78 (3.22)	
Post intervention	19.11 (3.24)	18.01 (3.22)	
Within-group change post intervention to baseline	0.72 (−3.22; 4.64)	−1.76 (−5.58; 2.06)	2.48 (−1.97; 6.93)
1W follow-up	19.38 (3.26)	19.41 (3.28)	
Within-group change 1W follow-up to baseline	0.99 (−3.03; 5.02)	−0.37 (−4.48; 3.74)	1.36 (−3.31; 6.03)

Abbreviations: NPRS, numeric pain rating scale: N, Newton. Data are mean ± standard error, frequency (proportion) or mean difference (95% confidence interval). * < 0.05.

## Data Availability

The data presented in this study are available on request from the corresponding author due to privacy restrictions.

## Supplementary Materials

The following supporting information can be downloaded at https://www.mdpi.com/article/10.3390/jcm14186619/s1, Table S1: Secondary outcome measures: headache-related and psychological parameters; Table S2: STRICTA 2010 checklist of information to include when reporting interventions in a clinical trial of acupuncture (Expansion of Item 5 from CONSORT 2010 checklist). Table S3: CONSORT 2010 checklist with the Non-pharmacological Trials Extension to CONSORT (with STRICTA 2010 extending CONSORT Item 5 for acupuncture trials).

## Abbreviations

The following abbreviations are used in this manuscript:

CeH	Cervicogenic headache
OCI	Obliquus capitis inferior
DN	Dry needling
SN	Sham needling
MT	Manual therapy
CFRT	Cervical flexion–rotation test
MD	Mean difference
CI	Confidence interval
TTH	Tension-type headache
ICHD	International classification of headache disorders
NP	Neck pain
JPE	Joint position error
ROM	Range of motion
PPT	Pressure pain threshold
STRICTA	Standards for reporting interventions in clinical trials of acupuncture
CONSORT	Consolidated standards of reporting trials
MDC	Minimal detectable change
MET	Muscle energy technique
SNAG	Sustained natural apophyseal glide
ICC	Intraclass correlation coefficient
CROM	Cervical range of motion device
CCFT	Craniocervical flexion test
NPRS	Numeric pain rating scale
MCID	Minimally clinical important difference
NDI	Neck disability index
HDI	Headache disability inventory
HIT-6	Headache impact test—6
GPES	Global perceived effect scale
CSI	Central sensitization inventory
PCS	Pain catastrophizing scale
TSK	Tampa scale for kinesiophobia
PCI	Pain coping inventory
BMI	Body mass index
Pc	Personal computer
Hrs	Hours
LMM	Linear mixed models
GEE	Generalized estimating equations
W	Week
ES	Effect size
BI	Bang’s blinding index

## Appendix C

Bang’s blinding index (BI) with 95% bootstrap confidence intervals. BI = 0.32 (95% CI: −0.40; 0.77) in the DN group and −0.29 (95% CI: −1.43; 0.36) in the SN group.

BI scores range from −1.00 (all participants incorrectly guessing the opposite intervention) to +1.00 (all participants correctly identifying their allocation), with 0.00 representing random guessing.

In this study, the DN group showed a positive BI value, while the SN group showed a negative value. This scenario is referred to as unblinded (DN)/opposite (SN). This indicates that patients in both groups tended to believe they had received the active treatment, regardless of their actual allocation, highlighting the influence of patient expectations and their preference for the presumed “true” intervention [67].

Data are frequency (proportion).

**Table A1:** Bang’s blinding index report.

	Guess	DN	SN	Do Not Know	Total
Intervention					
DN		8 (53.3%)	3 (20.0%)	4 (26.7%)	15
SN		5 (29.4%)	5 (29.4%)	7 (41.2%)	17
Total		13 (40.6%)	8 (25.0%)	11 (34.4%)	32
