# Supplementary material for: Effects of Dry Needling of the Obliquus Capitis Inferior in Patients with Cervicogenic Headache and Upper Cervical Dysfunction: An Exploratory Randomized Sham-Controlled Trial
Source: J Clin Med. 2025 Sep 19;14(18):6619. doi: 10.3390/jcm14186619 (PMC12471241; doi:10.3390/jcm14186619)
Supplement: Supplementary file 1 [file jcm-14-06619-s001.zip › jcm-3794025-supplementary.pdf]

Table S1. Secondary outcome measures: headache-related and psychological parameters.

**Headache-related parameters**			
Pain intensity			
MEAN PAIN SCORE PAST 7 DAYS (NPRS /10)			
**Baseline**	4,09 (0,55)	5,07 (0,52)	
**1W Follow-up**	3,93 (0,55)	4,43 (0,58)	
Within group change 1W follow-up to baseline	-0,16 (-1,27 ; 0,95)	-0,65 (-1,81; 0,52)	0,49 (-1,12 ; 2,09)
MAXIMAL PAIN SCORE PAST 7 DAYS (NPRS /10)			
**Baseline**	5,91 (0,56)	7,04 (0,55)	
**1W Follow-up**	5,49 (0,58)	6,26 (0,61)	
Within group change 1W follow-up to baseline	-0,42 (-1,63 ; 0,79)	-0,78 (-2,05; 0,48)	0,36 (-1,39 ; 2,11)
Disability & impact on daily functioning			
NDI (%)			
**Baseline**	25,00 (3,02)	30,94 (2,93)	
**1W Follow-up**	21,55 (3,07)	27,73 (3,13)	
Within group change 1W follow-up to baseline	-3,45 (-8,06 ; 1,17)	-3,21 (-8,10 ; 1,68)	-0.24 (-6,96 ; 6,48)
HDI (/100)			
**Baseline**	35,88 (4,62)	39,06 (4,48)	
**1W Follow-up**	35,46 (4,65)	31,88 (4,63)	
Within group change 1W follow-up to baseline	0,42 (-4,26 ; 5,10)	**-7,18* (-12,17 ; -2,18)**	6.76 (-0,086 ; 13,61)
HIT-6 (/78)			
**Baseline**	61,56 (1,56)	62,12 (1,51)	
**1W Follow-up**	58,15 (1,58)	58,68 (1,62)	
Within group change 1W follow-up to baseline	**-3,42* (-5,90 ; -0,93)**	**-3,44* (-6,07 ; -0,81)**	0.022 (-3,60 ; 3,64)
GLOBAL PERCEIVED EFFECT (GPES)			
1W follow-up, n (%)			
Slightly worsened	0/13 (0%)	2/13 (15,4%)	
No change	5/13 (38,5 %)	4/13 (30,8%)	
Slightly improved	8/13 (61,5 %)	4/13 (30,8%)	
Much improved	0/13 (0%)	3/13 (23,1%)	

**Psychological parameters**			
CENTRAL SENSITIZATION (CSI /100)			
**Baseline**	34,19 (3,37)	43,47 (3,27)	
**1W Follow-up**	34,58 (3,39)	42,64 (3,33)	
Within group change 1W follow-up to baseline	0,39 (-2,05 ; 2,84)	-0,83 (-3,45 ; 1,78)	1,23 (-2,35 ; 4,80)
CATASTROPHIZING (PCS /52)			
**Baseline**	16,56 (2,97)	21,47 (2,88)	
**1W Follow-up**	17,27 (3,02)	14,96 (3,09)	
Within group change 1W follow-up to baseline	0,71 (-4,06 ; 5,75)	-6,51 (-11,56 ; -1,47)	7,22 (0,28 ; 14,16)
KINESIOPHOBIA (TSK /68)			
**Baseline**	34,31 (1,81)	32,88 (1,76)	
**1W Follow-up**	33,72 (1,84)	29,50 (1,88)	
Within group change 1W follow-up to baseline	-0,59 (-3,47 ; 2,28)	-3,39 (-6,43 ; -0,34)	2,79 (-1,39 ; 6,98)
COPING STYLE (PCI)			
Baseline, n (%)			
Active coping	4/17 (23,5%)	7/16 (43,8%)	
Passive coping	13/15 (76,5%)	9/16 (56,3%)	
1W Follow-up, n (%)			
Active coping	6/15 (40%)	10/13 (76,9%)	
Passive coping	9/15 (60%)	3/13 (23,1%)	

Abbreviations: NPRS, numeric pain rating scale; NDI, neck disability index; HDI, Headache disability inventory; HIT-6, headache impact test-6; CSI, central sensitization inventory; PCS, pain catastrophizing scale; TSK, tampa scale of kinesiophobia; PCI, pain coping inventory. Data are mean ± standard error, frequency (proportion) or mean difference (95% confidence interval). ***=<0,05; **=<0,001**

Table S2: STRICTA 2010 checklist of information to include when reporting interventions in a clinical trial of acupuncture (Expansion of Item 5 from CONSORT 2010 checklist)

**Item**	**Detail**	**Page/line**
**1. Acupuncture rationale**	1a) Style of acupuncture (e.g. Traditional Chinese Medicine, Japanese, Korean, **Western medical**, Five Element, ear acupuncture, etc)	P4-5/143-153
	1b) Reasoning for treatment provided, based on historical context, literature sources, and/or consensus methods, with references where appropriate	P2/78-87
	1c) Extent to which treatment was varied	Not applicable
**2. Details of needling**	2a) Number of needle insertions per subject per session (mean and range where relevant)	P4-5/160-163
	2b) Names (or location if no standard name) of points used (uni/bilateral)	Not applicable
	2c) Depth of insertion, based on a specified unit of measurement, or on a particular tissue level	P4-5/142-163

	2d) Response sought (e.g. *de qi* or muscle twitch response)	P5/153
	2e) Needle stimulation (e.g. manual, electrical)	P4/143
	2f) Needle retention time	Not applicable
	2g) Needle type (diameter, length, and manufacturer or material)	P4/144
**3. Treatment regimen**	3a) Number of treatment sessions	P1/16
	3b) Frequency and duration of treatment sessions	Not applicable
**4. Other components of treatment**	4a) Details of other interventions administered to the acupuncture group (e.g. moxibustion, cupping, herbs, exercises, lifestyle advice)	P5/165-172
	4b) Setting and context of treatment, including instructions to practitioners, and information and explanations to patients	P5/160-163
**5. Practitioner background**	5) Description of participating acupuncturists (qualification or professional affiliation, years in acupuncture practice, other relevant experience)	P5/169
**6. Control or comparator interventions**	6a) Rationale for the control or comparator in the context of the research question, with sources that justify this choice	P5/155-157
	6b) Precise description of the control or comparator. If sham acupuncture or any other type of acupuncture-like control is used, provide details as for Items 1 to 3 above.	P5/153-160

Table S3: CONSORT 2010 checklist with the Non-pharmacological Trials Extension to CONSORT (with STRICTA 2010 extending CONSORT Item 5 for acupuncture trials)

Section/Topic	Item #	CONSORT 2010 Statement*: Checklist item[10]. Describe:	Additional items from the Non-pharmacological Trials Extension to CONSORT[14]. Add:	Page/line
TITLE AND ABSTRACT				
	1.a	Identification as a randomized trial in the title	In the abstract, description of the experimental treatment, comparator, care providers, centres and blinding status.	Tittle page
	1.b	Structured summary of trial design, methods, results, and conclusions; for specific guidance see CONSORT for Abstracts [58,59]		P1/13-34
INTRODUCTION				P2-3/39-95
Background and objectives	2.a	Scientific background and explanation of rationale		P2/50-88
	2.b	Specific objectives or hypotheses		P3/89-95
METHODS				P3-8/96-321
Trial design	3.a	Description of trial design (e.g., parallel, factorial) including allocation ratio		P3/103
	3.b	Important changes to methods after trial commencement (e.g. eligibility criteria), with reasons		Not applicable
Participants	4.a	Eligibility criteria for participants	When applicable, eligibility criteria for centers and those performing the interventions.	P3-4/109-124
	4.b	Settings and locations where the data were collected		P3/109-112
**Interventions**	**5**	**The interventions for each group with sufficient details to allow replication, including how and when they were actually administered**	**Precise details of both the experimental treatment and comparator - see Table 1 for details**	P4-5/142-172

Section/Topic	Item #	CONSORT 2010 Statement*: Checklist item[10]. Describe:	Additional items from the Non-pharmacological Trials Extension to CONSORT[14]. Add:	Page/line
Outcomes	6.a	Completely defined pre-specified primary and secondary outcome measures, including how and when they were assessed		P5-8/173-296
	6.b	Any changes to trial outcomes after the trial commenced with reasons		Not applicable
Sample size	7.a	How sample size was determined	When applicable, details of whether and how the clustering by care providers or centers was addressed.	P4/136-141
	7.b	When applicable, explanation of any interim analyses and stopping guidelines		Not applicable
Randomization				P4/125-135
Sequence generation	8.a	Method used to generate the random allocation sequence	When applicable, how care providers were allocated to each trial group.	P4/128-129
	8.b	Type of randomization; details of any restriction (e.g., blocking and block size)		P4/129
Allocation concealment	9	Mechanism used to implement the random allocation sequence (e.g., sequentially numbered containers), describing any steps taken to conceal the sequence until interventions were assigned		P4/130
Implementation	10	Who generated the random allocation sequence, who enrolled participants, and who assigned participants to interventions		P4/130

Section/Topic	Item #	CONSORT 2010 Statement*: Checklist item[10]. Describe:	Additional items from the Non-pharmacological Trials Extension to CONSORT[14]. Add:	Page/line
Blinding	11.a	If done, who was blinded after assignment to interventions (e.g. participants, care providers, those assessing outcomes) and how	Whether or not those administering co-interventions were blinded to group assignment. If blinded, method of blinding and description of the similarity of interventions.	P4/130-135 + appendix: blinding index.
	11.b	If relevant, description of the similarity of interventions		Not applicable
Statistical methods	12.a	Statistical methods used to compare groups for primary and secondary outcomes	When applicable, details of whether and how the clustering by care providers or centers was addressed.	P8/297-321
	12.b	Methods for additional analyses, such as subgroup analyses and adjusted analyses		Not applicable
RESULTS				P8-16/322-469
Participant flow (A diagram is strongly recommended)	13.a	For each group, the numbers of participants who were randomly assigned, received intended treatment, and were analyzed for the primary outcome	The number of care providers or centers performing the intervention in each group and the number of patients treated by each care provider or in each center.	P10/fig 1
	13.b	For each group, losses and exclusions after randomization, together with reasons		P10/fig 1
Recruitment	14.a	Dates defining the periods of recruitment and follow-up		P3/109
	14.b	Why the trial ended or was stopped		Not applicable
Baseline data	15	A table showing baseline demographic and clinical characteristics for each group	When applicable, a description of care providers (case volume, qualification, expertise, etc.) and centers (volume) in each group.	P9/table 2

Section/Topic	Item #	CONSORT 2010 Statement*: Checklist item[10]. Describe:	Additional items from the Non-pharmacological Trials Extension to CONSORT[14]. Add:	Page/line
Numbers analyzed	16	For each group, number of participants (denominator) included in each analysis and whether the analysis was by original assigned groups		P10/fig 1
Outcomes and estimation	17.a	For each primary and secondary outcome, results for each group, and the estimated effect size and its precision (e.g., 95% confidence interval)		P11-15/386-458
	17.b	For binary outcomes, presentation of both absolute and relative effect sizes is recommended		Not applicable
Ancillary analyses	18	Results of any other analyses performed, including subgroup analyses and adjusted analyses, distinguishing pre-specified from exploratory		Not applicable
Harms	19	All important harms or unintended effects in each group; for specific guidance see CONSORT for Harms [60]		P16/459-469
DISCUSSION				P16-19/ 470-620
Limitations	20	Trial limitations, addressing sources of potential bias, imprecision, and, if relevant, multiplicity of analyses		P18/590-605

Section/Topic	Item #	CONSORT 2010 Statement*: Checklist item[10]. Describe:	Additional items from the Non-pharmacological Trials Extension to CONSORT[14]. Add:	Page/line
Generalizability	21	Generalizability (external validity, applicability) of the trial findings	Generalizability (external validity) of the trial findings according to the intervention, comparators, patients and care providers and centers involved in the trial.	P19/610-613
Interpretation	22	Interpretation consistent with results, balancing benefits and harms, and considering other relevant evidence	In addition, take into account the choice of the comparator, lack of or partial blinding, unequal expertise of care providers or centers in each group.	P19/606-626
OTHER INFORMATION				
Registration	23	Registration number and name of trial registry		P3/97-101
Protocol	24	Where the full trial protocol can be accessed, if available		Not applicable
Funding	25	Sources of funding and other support (e.g., supply of drugs); role of funders		Not applicable
